# Exploring hospital-based health information technology functions for patients with Alzheimer’s Disease and related Dementias

**DOI:** 10.1016/j.pmedr.2021.101459

**Published:** 2021-06-23

**Authors:** Nianyang Wang, Asmaa Albaroudi, Ivy Benjenk, Jie Chen

**Affiliations:** Department of Health Policy and Management, University of Maryland, School of Public Health, College Park, MD, USA

**Keywords:** Health information technology, Alzheimer’s Disease and Related Dementias, Care coordination, Preventable hospitalizations, Continuity of patient care, ADRD, Alzheimer's Disease and Related Dementias, AHAIT, American Hospital Association Annual Survey Information Technology Supplement, AHRF, Area Health Resources File, ED, Emergency Department, HIE, Health Information Exchange, HIT, Health Information Technology, PCP, Primary care provider, SEDD, State Emergency Department Databases

## Abstract

This study investigated whether hospital-adopted health information technology (HIT) is associated with a reduction in the frequency of preventable emergency department (ED) visits for patients with Alzheimer’s Disease and Related Dementias (ADRD). We used data from the 2015 State Emergency Department Databases, Area Health Resources File, and the American Hospital Association Annual Survey Information Technology Supplement. We employed multivariable logistic regression models to examine the variation of the likelihood of having preventable ED visits by hospitals’ adoption of HIT functions and adjusted for patient, hospital, and county-level factors. We focused on hospital-HIT functions related to patient engagement, routine integration and availability of electronic clinical information, frequency of hospital reported use of electronic patient information, and the provision of electronic notification to the patient's primary care provider. Approximately 23% of ADRD patients went to a hospital that often used electronic records from outside providers, and 75% of ADRD patients went to a hospital that provided electronic notification to the patient's primary care provider. Regression results showed that hospital reported use of electronic patient health information from outside providers (OR = 0.88; p < 0.001), provision of electronic notification to the patient's primary care physician inside and outside of the system (OR = 0.91; p = 0.013), and hospital-HIT patient engagement functionalities (OR = 0.90; p < 0.001) were associated with significantly lower preventable ED visit rates. The results of our study suggest that certain types of HIT functionalities may be useful for reducing preventable ED visits for ADRD patients.

## Highlights

• The study focused on five care coordination components of HIT among ADRD patients.
• HIT patient engagement linked to a significant reduction in preventable ED visits.
• Certain types of HIT are useful for reducing preventable EDs for ADRD patients.
• HIT can improve the wellbeing of ADRD patients especially during the COVID crisis.

## 1 Introduction

Older adults with Alzheimer’s disease and related dementia (ADRD) have substantially higher rates of emergency department (ED) use than older adults without ADRD, with 47% of adults with ADRD having at least one ED visit annually (Amjad et al., 2016). In addition to the high rate of ED utilization, persons with ADRD also have high rates of ED revisits. The ED revisit rate in this population has been found to be 6–20 percentage-points greater than the revisit rate for older adults without dementia and this difference has been found to be significant after adjusting for health and demographic factors (LaMantia et al., 2016, Kent et al., 2019), Thirty-day ED revisits are often deemed preventable and are often attributed to ineffective care coordination and continuity of care (Han et al., 2015).

Health information technologies (HIT) are increasingly being used by hospitals to promote HIT care coordination, which is defined as the use of HIT to enable both providers and patients to better coordinate transitions of care, especially follow-up care after going to the ED (Foster and Krasowski, 2019, Martínez Nicolás et al., 2019, Rahurkar et al., 2021). Care coordination may be successfully achieved through the use of data sharing between hospitals and outpatient providers through portals (The Office of the National Coordinator for Health Information Technology (ONC), 2017), health information exchanges (HIEs) (The Office of the National Coordinator for Health Information Technology (ONC), 2020), using interfaces to integrate the HIE into the electronic medical record (Integration, 2018), and automatic notifications regarding care transitions (Office of the National Coordinator for Health Information Technology, 2013). The integration of clinical data into the ED electronic health record allows providers to make informed treatment decisions and facilitates discharge planning (Poku et al., 2019). HIEs allow for the dissemination of ED and hospital records to community-based providers and aim to minimize the communication failures that frequently occur during the care transition process (Vest et al., 2015, Chen et al., 2019). Automatic notifications of ED visits and inpatient admissions and discharges to primary care providers encourage timely follow-up and have been found to decrease the risk of readmissions among Medicare fee-for-service beneficiaries (Unruh et al., 2017).

In addition, hospital-based HIT can be used to advance HIT patient engagement functionalities, which is defined as the use of hospital-based HIT functionalities for the active involvement of patients and their caregivers (Asagbra et al., 2019). This includes the effective design of patient portals, which can allow patients to view their medical records, schedule appointments, and communicate with their providers through a platform that they can use on their computer or smartphone (Irizarry et al., 2015). Hospitals that report higher rates of adoption of patient engagement functionalities have been found to have lower readmission rates and higher patient satisfaction rates (Asagbra et al., 2019, Elysee et al., 2021). Patient portals have specifically been found to improve adherence to medications, reduce medical errors, and improve patient-provider communication (Dendere et al., 2019).

This study aims to illustrate the association between hospital-based HIT care coordination strategies and hospital-based HIT patient engagement strategies to the rate of preventable ED visits for older adults with ADRD, of which there is limited literature. We hypothesize that hospital-based HIT functionalities, especially those that can promote communication and information exchanges among patients/caregivers and their providers, can lead to reduced preventable ED visits for ADRD patients who usually have complex health needs. In particular, a well-designed HIT infrastructure can facilitate the information exchange among a team of providers (e.g., primary care and hospitals) and advance care coordination (Dixon et al., 2018). In addition, a patient-centered HIT patient portal can engage and empower patients and their caregivers. For example, an easy access patient portal can encourage patients and their caregivers to ask clarifying questions, communicate with providers, and transfer information among providers (Dixon et al., 2018). As such, we are more likely to observe efficient care management and treatment plans for ADRD patients (e.g., lower rates of preventable ED visits) under an effective HIT-facilitated information exchange platform. This study combines patient-level, hospital-level, and county healthcare data to use a more comprehensive data set. We measure the association between HIT care coordination and patient engagement strategies and preventable ED visits.

## 2 Methods

### 2.1 Data

Our study merges patient-level data from the 2015 State Emergency Department Databases (SEDD), county-level data from the 2015 Area Health Resources File (AHRF), and hospital-level organizational and HIT data from the 2015 American Hospital Association Annual Survey Information Technology Supplement (AHAIT). The SEDD consists of all discharge data from the emergency department visits for a state in the given year. We used data from Arizona, Florida, Kentucky, Maryland, North Carolina, Vermont, and Wisconsin due to the availability of necessary variables such as patients’ race/ethnicity and linkage to hospital and county data.

### 2.2 Population

We used ICD-9 codes in Quarters 1–3 and ICD-10 codes in Quarter 4 to identify the ADRD diagnosis. ICD-9 and ICD-10 codes for any ADRD diagnosis code (primary, secondary, etc.) were identified from the Alzheimer’s Association and previously published studies (Alzheimer’s Association, 2018, Alzheimer’s Association Cognitive Impairment Care Planning Toolkit, 2018).

### 2.3 Dependent variable: preventable emergency department visits

We measured preventable ED visits by applying the New York University (NYU) ED visit algorithm onto the primary diagnoses for each patient, which classifies each ED visit with a probability of ED care being potentially preventable if timely ambulatory care had been provided based on the patient’s primary diagnoses (NYU, 2016, Ballard and Mary, 2010). We produced the probability of each patient having an ED visit that is needed but was potentially preventable/avoidable if timely and proper ambulatory care had been provided based on the primary diagnosis and used a threshold of ≥ 50% to establish if the ED visit was preventable based on existing literature (Guthrie et al., 2017).

### 2.4 Health IT measures

The key independent variables are HIT measures we obtained from the AHAIT. We created 5 components of hospital-adopted HIT in this study (Table 1): (1) Routine integration of electronically received clinical information from outside sources, (2) Routinely have necessary clinical information available electronically from outside providers, (3) Often use hospital reported electronic patient health information from outside providers, (4) Provide electronic notification to the patient's PCP, and (5) HIT patient engagement. Patient engagement is important since patients in the early stages of ADRD may benefit from having access to a patient portal as it will allow them to reference their medication lists and ask providers questions after they are discharged from the hospital. For patients with more advanced ADRD, this technology can be utilized by their caregivers. The portal may be able to give them a virtual opportunity to review clinical information, confirm that their family member is receiving the correct medications while in the hospital, and help facilitate adherence to the discharge plan. Hospitals responded to nine indicators of HIT patient engagement. We calculated the summation of the indicators of patient engagement HIT functionalities that the hospital adopted (Asagbra et al., 2019). We further dichotomized the number of adopted functionalities into low engagement which was defined as having 0–7 (below the median) total functionalities, and high engagement was defined as having 8–9 (above the median) total functionalities present to evenly divide the hospitals and show differences in HIT patient engagement level. Sensitivity analyses (see below) were used to test different measures of the HIT patient engagement indices.

**Table 1:** Sample Characteristics of Hospital Health Information Technology for Patients with ADRD.

Variable	Frequency	Percent
Preventable ED Visit		
Not Preventable	102,714	94.4
Preventable	6,114	5.6
Hospital-based HIT adoption		
1. Routinely Integrate Electronic Clinical Information from Outside Sources		
Yes	33,695	31.0
No	75,133	69.0
2. Routinely Have Clinical Information Available Electronically from Outside Providers		
Yes	70,767	65.0
No	35,703	32.8
Don’t Know	2,358	2.2
3. Often Use Electronic Patient Health Information from Outside Providers		
Yes	25,466	23.4
No	83,362	76.6
4. Provide Electronic Notification to the Patient's Primary Care Provider		
Only Inside System	37,440	34.4
Inside and Outside System	44,358	40.8
Don’t Notify/Don’t Know	27,030	24.8
5. Health Information Technology Patient Engagement Functionalities		
Below Median/Low Engagement Functionalities (0–7)	52,879	48.6
Above Median/High Engagement Functionalities (8–9)	55,949	51.4
*Individual Patient Engagement Functionalities*		
Patient can view information from their health/medical record online		
Yes	108,048	99.3
No	780	0.7
Patient can download information from their health/medical record		
Yes	101,728	93.5
No	7,100	6.5
Patient can electronically send care/referral summaries to a third party		
Yes	90,323	83.0
No	18,505	17.0
Patient can request an amendment to change/update their health/medical record		
Yes	93,507	85.9
No	15,321	14.1
Patient can request refills for prescriptions online		
Yes	57,294	52.6
No	51,534	47.4
Patient can schedule appointments online		
Yes	64,739	59.5
No	44,089	40.5
Patient can pay bills online		
Yes	98,124	90.2
No	10,704	9.8
Patient can exchange secure messages with their provider		
Yes	61,454	56.5
No	47,374	43.5
Patient can submit self-generated data		
Yes	82,556	75.9
No	26,272	24.1

Note. Sample size: 108,828. Data sources: 2015 SEDD, AHA Annual Survey Information Technology Supplement, and Area Health Resources File. Our sample consists of seven states (Arizona, Florida, Kentucky, Maryland, North Carolina, Vermont, and Wisconsin) and is comprised of patients with ADRD and had complete data. Percentages may not add up to 100% due to rounding. All AHAIT questions were asked to all hospitals.

### 2.5 Covariates

We chose our covariates based on the ADRD literature as well as the Andersen Healthcare Utilization Model (Andersen, 1995, Lyketsos and Olin, 2002, Wang et al., 2021) and controlled for race, gender, age, insurance, zip code income quartile, Elixhauser comorbidities index (a quantitative measure of patient’s disease burden based on existing comorbidities using ICD diagnosis codes, a high Elixhauser score is indicative of a higher risk of mortality (van Walraven et al., 2009)), last quarter index, county urban/rural status, county percent African American, county Health Professional Shortage Area (HPSA) status, county Mental Health Professional Shortage Area (MHPSA) status, hospital number of beds, hospital ownership type, and state fixed effects. The county-level measures were selected based on literature that shows higher rates of preventable ED visits for ADRD patients in healthcare provider shortage areas (Wang et al., 2020).

### 2.6 Data analysis

We first presented the definitions and descriptive statistics of hospital-based HIT measures. Characteristics of the study sample were included in the supplement. We used a two-sided p-value of 0.05 to determine the significance level. We applied a multivariable logistic regression to test the association between preventable ED visits with each of the HIT variables, controlling for the covariates to examine the adjusted odds ratio of the specified HIT component. Data analyses were performed using Stata version 15.

We performed additional sensitivity analyses to make sure that our results were robust. Specifically, we (1) tested our model among patients with routine discharges (vs. discharges to skilled nursing facilities and long term care facilities); (2) used different cutoffs for the percentage threshold of preventable ED visits based on the NYU algorithm (40% and 60%); and (3) examined different measures of HIT patient engagement functionalities. In our main analysis, we based our dichotomization of patient engagement assuming that all functionalities are equivalent in regards to relevance (Asagbra et al., 2019). In the sensitivity analysis, we used continuous (0–9) and quartiles (0–5, 6–7, 8, 9) measures, and also tested individual HIT patient engagement features.

## 3 Results

Our final sample size of 108,828 was established based on patients with ADRD with no missing data, allowing us to merge among the three datasets. Over 65% of ADRD patients in our sample went to a hospital that had an EHR system that routinely had clinical information available electronically from outside providers (Table 1). However, only 31% went to a hospital that routinely integrated electronic clinical information from outside sources and only 23% went to a hospital that reported frequent use of electronic patient health information from outside providers. Forty-one percent went to a hospital that provided electronic notification to the patient's PCP whether the PCP was inside or outside the hospital’s system.

Table 2 presented the adjusted odds ratio for each HIT component. Often using electronic patient health information from outside providers was negatively associated with the likelihood of preventable ED rates (OR = 0.88, p < 0.001). The adjusted odds ratio of the integration of electronically received clinical information from outside sources or the availability of electronic clinical information from outside providers were not significant. Hospitals that electronically notify the patient's PCP regardless of whether they were in the system or not were also negatively associated with the likelihood of having a preventable ED compared to hospitals that only notify if inside the system (OR = 0.91, p = 0.013). A high level of HIT patient engagement functionalities (8–9 functions) was negatively associated with the likelihood of preventable ED visits compared to low HIT patient engagement functionalities (OR = 0.90, p < 0.001). A table showing the full regression results for patient engagement with all adjusted variables is available in the supplementary file.

**Table 2:** Adjusted Logistic Regressions of the Association Between Health Information Technology Components and Preventable Emergency Department Visits for ADRD Patients.

Variable	OR	95% CI	p-value
Health Information Technology Patient Engagement Functionalities			
Low Engagement Functionalities (0–7)	Ref		
High Engagement Functionalities (8–9)	0.90	0.85–0.95	<0.001
Individual HIT-Patient Engagement Function			
Patient can electronically send care/referral summaries to a third party	0.89	0.83–0.96	0.002
Patient can request an amendment to change/update their health/medical record	0.85	0.79–0.92	<0.001
Patient can request refills for prescriptions online	0.89	0.84–0.95	<0.001
Patient can submit self-generated data	0.93	0.87–0.99	0.031
Routinely Integrate Electronic Clinical Information from Outside Sources			
No	Ref		
Yes	1.05	0.99–1.12	0.121
Routinely Have Clinical Information Available Electronically from Outside Providers			
No	Ref		
Yes	0.98	0.92–1.04	0.550
Don’t Know	1.03	0.85–1.25	0.743
Often Use Electronic Patient Health Information from Outside Providers			
No	Ref		
Yes	0.88	0.82–0.95	<0.001
Provide Electronic Notification to the Patient's Primary Care Physician			
Only Inside System	Ref		
Inside and Outside System	0.91	0.84–0.98	0.013
Don't Notify/Don't Know	1.03	0.95–1.10	0.501

Notes. Abbreviations: OR = adjusted odds ratio, CI = confidence interval. These are results from four separate regressions. Each estimation function adjusted for patients’ race, gender, age, insurance, zip code income quartile, Elixhauser comorbidities, county urban/rural status, county percent African American, county HPSA status, county MHPSA status, hospital number of beds, hospital ownership, last quarter index, and state fixed effects. Sensitivity tests were performed based on different cutoffs for preventable ED visits (40% and 60%). Sample size: 108,828. Full results are available upon request.

Furthermore, the results of our sensitivity analyses were consistent. In Table 1, we also presented associations between preventable ED visits and specific hospital-based HIT patient engagement, and four patient engagement features (i.e., patients can electronically send care summaries to a third party, request an amendment to update their health record, request refills for prescription online, and submit self-generated data) had significant associations.

## 4 Discussion

Our study adds to the literature by demonstrating that hospitals which use HIT care coordination and patient engagement strategies were associated with lower odds of preventable ED visits for patients with ADRD compared with hospitals that did not use HIT for care coordination. The results showed that frequent hospital reported use of electronic patient health information from outside providers and electronic notification to a patient’s PCP were linked to lower preventable ED visit rates. Many patients, especially those with ADRD, may struggle to provide comprehensive health histories during hospitalizations. When hospital-based providers have access to clinically meaningful outside medical records (including baseline laboratory values and diagnostic history) that are well-organized and integrated into their own electronic health record system, they can use this information to guide their differential diagnosis and treatment planning decisions (Gordon et al., 2015).

We find that while 65% of hospitals receive clinical data from outside sources, only 23% of hospitals often use health information received electronically from outside providers or sources when treating patients. More research is needed to understand how to encourage and engage health care providers to routinely review the data and evaluate the overall patient health needs and comprehensive health care utilization. Our speculation is that the timeliness of data, completeness of patient records, and ease of access can all contribute to the frequency of data use. Policy initiatives and systematic efforts are likely needed to improve data interoperability and availability, including improved interfaces between EHRs and HIEs.

As compared to hospitals that only alert primary care providers in their health system when a patient is admitted or discharged, we found that hospitals that also notify providers who are outside of the healthcare system have even lower odds of preventable ED visits for ADRD patients. This adds to the evidence that receiving notifications related to patient discharge allows for improvements in care coordination (Unruh et al., 2017). Notifying a patients' PCP is essential, given that the notification would likely help support continuity of care (McMillan et al., 2013).

In addition to provider-focused HIT functionalities, our study suggested the importance of adopting patient-centered HIT functionalities. Specifically, we found an association between higher numbers of HIT patient engagement functionalities with a reduced likelihood of preventable ED visits for ADRD patients across U.S. hospitals. These functionalities can allow persons with ADRD and their caregivers to be able to review healthcare records and discharge information from the hospitalization to ensure that the patient is undergoing the proper post-discharge care and also contact providers and schedule appointments if needed. Individually, we found that among the patient engagement functionalities, that the ability for the patient to electronically send care/referral summaries to a third party, request an amendment to change/update their health/medical record, request refills for prescriptions online, and submit self-generated data are significantly associated with less preventable ED visits. As such, the functionalities of whether patients can view information from their health/medical record online, download information from their health/medical record, schedule appointments online, pay bills online, and exchange secure messages with their provider were not significant. These are engagement strategies that support successful transitions of care and successful management of chronic conditions in the community. Physicians can virtually monitor and proactively intervene in the care of large panels of patients with the use of patient-generated health data (e.g., exercise, blood sugars, weights) (Zhou et al., 2010).

Patients with ADRD may not have the cognitive ability or technological skills to engage in HIT patient engagement functions. Patients with early stages of ADRD could potentially find value in a patient portal, which may provide them ongoing access to their medication lists as well as provide an outlet for patients to submit questions to providers post-hospital discharge, for example. Individuals with advanced ADRD may rely on their caregivers to access the portal. Caregivers can use the portal to remotely stay abreast of their loved one’s clinical record, confirm their loved one is being provided the right medication in the hospital, and help support adherence to the care plan. Although patient portals can be difficult to use and <20% of patients have been found to use patient portals within 30-days of hospital discharge, patients who are older and have more chronic conditions and their caregivers have been found to be more likely to use patient portals after discharge (Griffin et al., 2016). A recent study also shows that after being taught how to navigate a patient portal, patients/caregivers report increased satisfaction as they do not need to wait to hear from the physician about a laboratory result or new medication orders (McAlearney et al., 2019). Although our study did not measure patients’ actual use of a patient portal, the results of our study shed light on the importance of designing a real-time patient-centered portal that can encourage bi-directional communication between patients/caregivers and their providers.

Our study had several limitations. First, our measures of the HIT functionalities are obtained from the AHA survey. We can only observe the overall system-level HIT without knowing the specific HIT features that are being applied to specific clinical diseases. The assumption of our study is that patients who were treated in hospitals with an effective patient information exchange system, for example, had an equal chance to benefit from this system. Hence, our study only explores the variation and can’t establish the causality of the use of certain HIT features on patients with certain diseases like ADRD. Future studies should facilitate specialized patient engagement strategies to design the care management and treatment strategy for people with complex health needs. Second, the HIT features we employed are systematic measures from perceptions of health care providers. We are not able to observe the actual use of patient portals by patients and their caregivers. In other words, the capability of patients to submit self-generated data does not mean that patients actually submitted self-generated data. Third, we cannot distinguish the actual use of patient portals among ADRD patients and their caregivers. Older adults with ADRD have a number of sensory deficits and many have limited digital literacy and access—as such, their caregivers might be the representatives and advocates for their health care needs and are the ones who use the HIT system (Brenowitz et al., 2019). Finally, our data did not contain other quality measures and modifiable factors. We advocate for more comprehensive HIT data collection and future studies can go in-depth to research other related factors.

## 5 Conclusion

The results of our study suggest that a well-designed HIT infrastructure that promotes communication and information exchange among multiple health care providers and ADRD patients/caregivers is associated with reduced preventable ED visits. Hospitals may consider increasing resources towards onboarding patients and their caregivers to the portal and working to enhance their regional HIEs, which can improve the overall health of patients given improvements in information exchange among providers. Further research is needed to identify the optimal care coordination effects of HIT for people with ADRD and complex health needs.

## Funding

This research was supported by the National Institutes of Health, National Institute on Minority Health and Health Disparities Grant 3R01MD011523-03S1; National Institutes of Health, National Institute on Aging Grant 1R01AG062315-01A1 (Chen, PI).

## CRediT authorship contribution statement

**Nianyang Wang:** Conceptualization, Data curation, Formal analysis, Writing - original draft, Writing - review & editing. **Asmaa Albaroudi:** Writing - review & editing, Validation, Investigation. **Ivy Benjenk:** Writing - review & editing, Methodology, Investigation. **Jie Chen:** Writing - review & editing, Methodology, Funding acquisition, Conceptualization.

## Declaration of Competing Interest

The authors declare that they have no known competing financial interests or personal relationships that could have appeared to influence the work reported in this paper.
