# Supplementary material for: Exploring hospital-based health information technology functions for patients with Alzheimer’s Disease and related Dementias
Source: Prev Med Rep. 2021 Jun 23;23:101459. doi: 10.1016/j.pmedr.2021.101459 (PMC8256283; doi:10.1016/j.pmedr.2021.101459)
Supplement: Supplementary Data 1 [file mmc1.docx]

**Supplement: Table 3**

Logistic Regression Results of the Association Between Health Information Technology Patient Engagement Functionalities and Preventable Emergency Department Visits for ADRD Patients

| Variable | OR | 95% CI | p-value |
| --- | --- | --- | --- |
| Health Information Technology Patient Engagement Functionalities |  |  |  |
| Low Engagement Functionalities (0-7) | Ref |  |  |
| High Engagement Functionalities (8-9) | 0.90 | 0.85-0.95 | <0.001 |
| Race |  |  |  |
| Non-Hispanic White | Ref |  |  |
| Non-Hispanic Black | 1.23 | 1.14-1.33 | <0.001 |
| Hispanic | 1.12 | 1.00-1.24 | 0.045 |
| Non-Hispanic Asian/Pacific Islander | 0.91 | 0.61-1.36 | 0.658 |
| Non-Hispanic Native American | 1.15 | 0.81-1.65 | 0.427 |
| Non-Hispanic Other | 0.98 | 0.71-1.37 | 0.921 |
| Gender |  |  |  |
| Male | Ref |  |  |
| Female | 0.92 | 0.87-0.97 | 0.002 |
| Age |  |  |  |
| 30-49 years old | Ref |  |  |
| 50-64 years old | 1.25 | 0.91-1.72 | 0.167 |
| 65-74 years old | 1.05 | 0.77-1.44 | 0.747 |
| 75+ years old | 0.97 | 0.71-1.32 | 0.842 |
| Primary Payer (Insurance) |  |  |  |
| Medicare | Ref |  |  |
| Medicaid | 0.98 | 0.81-1.19 | 0.829 |
| Other | 0.99 | 0.90-1.08 | 0.763 |
| Zip Code Income Percentile |  |  |  |
| 0-25th percentile | Ref |  |  |
| 26th-50th percentile | 1.03 | 0.96-1.10 | 0.431 |
| 51st-75th percentile | 1.00 | 0.92-1.08 | 0.991 |
| 76th-100th percentile | 0.97 | 0.88-1.06 | 0.502 |
| Elixhauser Comorbidities |  |  |  |
| ≤2 | Ref |  |  |
| >2 | 2.87 | 2.72-3.02 | <0.001 |
| Time Index (Quarter of Year 2015) |  |  |  |
| Q1-Q3 | Ref |  |  |
| Q4 | 1.04 | 0.98-1.10 | 0.219 |
| Urban/Rural |  |  |  |
| Urban | Ref |  |  |
| Rural | 1.33 | 1.22-1.45 | <0.001 |
| County Percent African American |  |  |  |
| Below Median Average (<13.9%) | Ref |  |  |
| Above Median Average (≥13.9%) | 0.99 | 0.93-1.06 | 0.819 |
| County Health Professional Shortage Area |  |  |  |
| Not/Part of County HPSA | Ref |  |  |
| Whole County HPSA | 0.92 | 0.78-1.08 | 0.300 |
| County Mental Health Professional Shortage Area |  |  |  |
| Not/Part of County Mental Health HPSA | Ref |  |  |
| Whole County Mental Health HPSA | 1.10 | 1.02-1.19 | 0.017 |
| Hospital Number of Beds |  |  |  |
| <200 | Ref |  |  |
| 200-299 | 1.05 | 0.97-1.14 | 0.224 |
| 300-499 | 0.96 | 0.89-1.05 | 0.385 |
| 500+ | 0.89 | 0.82-0.96 | 0.003 |
| Hospital Ownership |  |  |  |
| For-profit | Ref |  |  |
| Not-for-profit | 0.95 | 0.86-1.04 | 0.274 |
| Government | 1.09 | 0.98-1.22 | 0.118 |
| States |  |  |  |
| Florida | Ref |  |  |
| Arizona | 1.03 | 0.87-1.22 | 0.756 |
| Kentucky | 1.21 | 1.09-1.34 | <0.001 |
| Maryland | 1.08 | 0.96-1.21 | 0.191 |
| North Carolina | 0.96 | 0.88-1.05 | 0.363 |
| Vermont | 1.29 | 0.85-1.96 | 0.231 |
| Wisconsin | 1.11 | 1.00-1.23 | 0.047 |

Notes. Abbreviations: OR = adjusted odds ratio, CI = confidence interval. Sample size: 108,828.
